# Hydraulic forces contribute to left ventricular diastolic filling

**DOI:** 10.1186/1532-429X-17-S1-P79

**Published:** 2015-02-03

**Authors:** Elira Maksuti, Marcus Carlsson, Håkan Arheden, Michael Broomé, Martin Ugander

**Affiliations:** 1 Department of Medical Engineering, School of Technology and Health, KTH Royal Insititute of Technology, Stockholm, Sweden; 2 Department of Clinical Physiology, Clinical Sciences, Lund University Hospital, Lund, Sweden; 3 Department of Physiology and Pharmacology, Karolinska Institute, Stockholm, Sweden; 4 Department of Clinical Physiology, Karolinska Institute and Karolinska University Hospital, Stockholm, Sweden

## Background

The mechanisms and forces involved in left ventricular diastolic filling are not completely known. The atrioventricular valve plane displacement (AVPD) can be seen as a piston moving in the apex-base direction. A hydraulic force (F) contributes to the movement of this piston during diastole according to the formula F = (Ventricular Area x Ventricular Pressure) - (Atrial Area x Atrial Pressure). Thus, a hydraulic force can contribute to the diastolic return of the atrioventricular plane if ventricular and atrial areas are different, even if the ventricular and atrial pressures are equal. Due to the irregular shape of the left atrium, Atrial Area is challenging to measure by planimetry. Therefore, we sought to indirectly calculate the Atrial Area from measurements of AVPD and left atrial filling. We hypothesized that the Atrial Area is smaller than the Ventricular Area, thus providing the basis for a hydraulic force in the apex-base direction during diastolic filling.

## Methods

Healthy volunteers (n=11, 5 women, age 24±3 years) underwent 1.5T cardiac magnetic resonance including left ventricular cine, and velocity-encoded phase contrast flow in the aorta and pulmonary veins. The Ventricular Area was defined as the largest short-axis epicardial area of the left ventricle at the level of AVPD, in accordance with prior validation from studies of systole (Carlsson M, et al, Am J Physiol Heart Circ Physiol, March 2007). AVPD was measured as the average of six locations from three long-axis planes. Ventricular ejection was defined as the time between the R-wave of the electrocardiogram and the first instance of negative aortic flow. Atrial filling during ventricular ejection (AFVE) was measured as the sum of the flow in all pulmonary veins during ventricular ejection. The Atrial Area was calculated as AFVE/AVPD.

## Results

The Atrial Area was smaller than the Ventricular Area (35.8±8.0 cm2 vs 43.6±8.0 cm2, p<0.001, mean difference 7.8 cm2 (17%) range 0.4-21.9 cm2 (1-49%)). AVPD was 16.6±1.8 mm and AFVE was 59.3±14.5 mL.

## Conclusions

A difference in area between the ventricle and atrium proves that a hydraulic force acts on the atrioventricular plane during left ventricular diastolic filling despite similar pressures in the ventricle and atrium. This force assists the apex-base movement of the atrioventricular plane as a consequence of cardiac anatomy. Further investigation is needed to evaluate the magnitude and significance of the hydraulic force in relation to other mechanisms contributing to diastolic filling such as elastic recoil and active relaxation.

## Funding

N/A.

